# Nitric Oxide Synthase Enzymes in the Airways of Mice Exposed to Ovalbumin: NOS2 Expression Is NOS3 Dependent

**DOI:** 10.1155/2010/321061

**Published:** 2010-10-05

**Authors:** Jennifer M. Bratt, Keisha Williams, Michelle F. Rabowsky, Michael S. Last, Lisa M. Franzi, Jerold A. Last, Nicholas J. Kenyon

**Affiliations:** Pulmonary and Critical Care Medicine, School of Medicine, Genome and Biomedical Sciences Facility, Suite 6500, 451 Health Sciences Dr., University of California, Davis, CA 95616, USA

## Abstract

*Objectives and Design*. The function of the airway nitric oxide synthase (NOS) isoforms and the lung cell types responsible for its production are not fully understood. We hypothesized that NO homeostasis in the airway is important to control inflammation, which requires upregulation, of NOS2 protein expression by an NOS3-dependent mechanism. *Materials or Subjects*. Mice from a C57BL/6 wild-type, NOS1^−/−^, NOS2^−/−^, and NOS3^−/−^ genotypes were used. All mice strains were systemically sensitized and exposed to filtered air or ovalbumin (OVA) aerosol for two weeks to create a subchronic model of allergen-induced airway inflammation. *Methods*. We measured lung function, lung lavage inflammatory and airway epithelial goblet cell count, exhaled NO, nitrate and nitrite concentration, and airway NOS1, NOS2, and NOS3 protein content. *Results*. Deletion of NOS1 or NOS3 increases NOS2 protein present in the airway epithelium and smooth muscle of air-exposed animals. Exposure to allergen significantly reduced the expression of NOS2 protein in the airway epithelium and smooth muscle of the NOS3^−/−^ strain only. This reduction in NOS2 expression was not due to the replacement of epithelial cells with goblet cells as remaining epithelial cells did not express NOS2. NOS1^−/−^ animals had significantly reduced goblet cell metaplasia compared to C57Bl/6 wt, NOS2^−/−^, and NOS3^−/−^ allergen-exposed mice. *Conclusion*. The airway epithelial and smooth muscle cells maintain a stable airway NO concentration under noninflammatory conditions. This “homeostatic” mechanism is unable to distinguish between NOS derived from the different constitutive NOS isoforms. NOS3 is essential for the expression of NOS2 under inflammatory conditions, while NOS1 expression contributes to allergen-induced goblet cell metaplasia.

## 1. Introduction

Nitric oxide (NO) plays multiple roles in the lung in both injury and repair; it is an airway and vascular smooth muscle cell signaling molecule, an inhibitory nonadrenergic noncholinergic (iNANC) signaling molecule, a modulator of apoptosis, and a component of the bactericidal arsenal of lung inflammatory cells. The primary molecular sources of NO are the NOS enzymes, including the inducible NOS2 isoform, which is upregulated in the ovalbumin- (OVA-) induced allergic airway inflammation model [1–3] and the constitutively expressed NOS1 (neuronal NOS) and NOS3 (endothelial NOS) isoforms, which contribute to the generation of NO in the murine airway epithelium [4, 5]. NO can be further metabolized to produce the more stable products, nitrate and nitrite. Both of these products are considered bioactive, capable of enzyme-dependent and enzyme-independent reconversion into NO [6, 7]. 

In asthma, increased NO concentration in exhaled breath is considered a disease biomarker and is supported by current guidelines for use in clinical settings [8]. The conventional treatment of asthmatics using inhaled steroids decreases exhaled NO [9], but whether alleviation of the asthmatic symptoms is a result of decreasing exhaled NO or if the decreased exhaled NO is a byproduct of decreasing overall lung inflammation is still debated. 

In the cell, maintenance of a stable NO concentration, or NO homeostasis, is essential, as fluctuations in the concentration of NO can alter intra and intercellular signaling and affect survivability. NO homeostasis, therefore, appears to be tightly regulated. Cook et al. [10], for example, detected an increase in the concentration of NO in exhaled breath correlating to an increase in NOS2 expression in NOS3^−/−^ mice, thus indicating a compensatory mechanism for the regulation of baseline NO production. Also, exposure of astrocytes to NO-scavenging hemoglobin increased NOS2 expression and was dependent on NF-*κ*B activation [11]. 

In addition to maintaining a baseline of NO production, it is necessary to upregulate NO production under specific conditions such as during oxidative stress [12], release of heme-containing compounds [13, 14], or the launching of an inflammatory response [15]. Thus, the ability to further regulate NOS2 expression in constitutive NOS isoform knockout strains may be necessary for the organism to modulate its reaction to an insult and also resolve the response. A study by Connelly et al. [16], using LPS stimulation, determined that NF-*κ*B translocation was necessary for NOS2 expression under inflammatory conditions and the study by Gobeil Jr. et al. identified NOS3 nuclearization as essential for NF-*κ*B activation leading to NOS2 expression [17]. 

We previously identified NOS2^−/−^ mice as being more susceptible to severe allergic inflammation and subepithelial fibrosis than their C57Bl/6 wild-type counterparts [1] indicating that NOS2 expression and activity are necessary for regulating the intensity of the inflammatory response. Thus, we chose to examine NO homeostatic dysregulation in the development of allergic airway disease. 

In this paper, we hypothesized that under normal conditions, deleting the NOS1 or NOS3 gene would upregulate NOS2 protein expression. Thus, the lung function and total cellular population of filtered air-exposed NOS1^−/−^ and NOS3^−/−^ mice should be comparable to filtered air-exposed C57Bl/6 wt mice, while the NOS2^−/−^ mice should have a heightened inflammatory cell profile as observed in our previous study [18]. Furthermore, Ten Broeke and colleagues showed that NOS2 expression was NOS3 dependent, and we therefore hypothesized that exposure of mice to ovalbumin (OVA) would inhibit this increase in NOS2 protein expression in the NOS3^−/−^ mice resulting in lower NOS2 protein concentrations compared to NOS3^−/−^ filtered air controls [19]. We also hypothesized that this would result in increased lung inflammation in the NOS3^−/−^ mice compared to both OVA-exposed C57Bl/6 wt and NOS1^−/−^ mice. To this end, we performed lung function analysis with a methacholine challenge protocol and collected bronchoalveolar lavage for the calculation of lung inflammatory cell influx and inflammatory cell profile. We also examined tissue-specific NOS2 expression patterns in the lungs and changes in nitric oxide production of both filtered air-exposed and OVA-exposed mice from NOS1^−/−^, NOS2^−/−^, NOS3^−/−^, and C57Bl/6 wt genotype.

## 2. Experimental Methods

### 2.1. Animals

All procedures were performed as per our IACUC-approved protocol, following their standards and regulations. All animals were maintained in an HEPA-filtered laminar flow cage rack with a 12-hour light/dark cycle and allowed free access to food and water. Animals were housed and cared for by the veterinary staff of Animal Resource Services at UCD in AALAC- accredited facilities, in plastic cages over autoclaved bedding in HEPA-filtered cage racks. Animals were routinely screened for health status by serology and histology by our veterinary animal resources facility. NOS1^−/−^, NOS3^−/−^, and the wild type strain C57BL/6 mice were purchased from Jackson Laboratories. The NOS2^−/−^ mice were initially purchased from Taconic Laboratories, Germantown, NY and were rederived by embryo transfer to establish a breeding colony in the Targeted Genomics Laboratory of the Mouse Biology barrier facility at UC Davis. They are on a C57BL/6 background and are designated C57BL/6Ai-[KO]*NOS2* N5 [20].

### 2.2. Exposure of Mice to OVA Aerosol

Mice were sensitized by intraperitoneal (ip) injection of chicken egg albumin (Ovalbumin, grade V, ≥98% pure, Sigma, St. Louis, MO, 2 × 10 *μ*g/0.1 mL, 2 weeks apart) with alum as an adjuvant [21]. Exposure to OVA aerosols was performed using chambers and generators we have described elsewhere [22]. Exposures to OVA aerosol, 10 mL of a 10 mg/mL (1%) solution, were begun on day 28. Mice were exposed for 30 minutes, three times per week for the duration of a given experiment. Age-matched control animals were injected ip with OVA (sensitized) but were then exposed to filtered air.

Histological evaluations of C57Bl/6 wt mice OVA-exposed and filtered air-exposed control mice demonstrated that we were able to induce airway inflammation, epithelial cell sloughing, and goblet cell hyperplasia in the exposed mice.

### 2.3. Lung Compliance and Resistance Measurements

Dynamic compliance and resistance of the respiratory system were measured using a plethysmograph for restrained animals. (Buxco Inc., Troy, NY). Mice were deeply anesthetized and sedated with medetomidine, 0.5 mg/kg (Domitor, Orion Pharma, Finland), and tiletamine/zolpidem, 50 mg/kg (Telazol, Fort Dodge Laboratories, Fort Dodge, IA) and ventilated at 7-8 cc/kg with a mouse ventilator (MiniVent, Harvard Apparatus, Cambridge, MA) for the duration of the procedure. Compliance and resistance measurements were made at baseline and immediately following serial 3-minute nebulizations of saline and methacholine (0, 0.5, 1.0, and 2.0 mg/mL).

### 2.4. Airway Inflammation

After the physiological measurements, animals were euthanized with an overdose of phenobarbital and dilantin administered via intraperitoneal injection. Animals were placed on a restraining board and their lungs lavaged with two 1 mL aliquots of phosphate-buffered saline (PBS), pH 7.4, and each aliquot was passaged twice through the lungs. Cells were pelleted at 2500 rpm for 10 minutes, and the acellular supernatant was removed and stored at −20°C for nitrate and nitrite (NO_x_) analysis. The remaining cell pellet was treated with a lysis buffer (0.15 M NH_4_Cl, 1 mM KHCO_3_, 0.1 mM EDTA, and pH 7.3), repelleted, and resuspended in 0.5 mL PBS. 

Total lavage cell number was determined using a hemocytometer and 100 *μ*L aliquots of the remaining cell suspension processed onto slides using a cytocentrifuge at 1650 rpm for 15 minutes. Slides were air dried and stained with a Hema3 stain set as described in the manufacturer's instructions (Biochemical Sciences, Swedesboro, NJ) and sealed using Cytoseal (Stephens Scientific, Kalamazoo, MI). Cell percent differentials were determined by counting 10 fields under a 40× objective. Cells were classified as alveolar macrophage, neutrophil, eosinophil, lymphocyte, or other based upon morphological characteristics and staining profile.

### 2.5. Measurement of Exhaled NO and NO_x _ Flux in Lung

Five-minute samples of exhaled gases were collected into a specially constructed Mylar bag from the cannulated mice via the ventilator exhalation port immediately after insertion of a mouse into the plethysmograph. This 5-minute sample was adequate for the measurement of NO concentration in the expired air, using a Sievers Nitric Oxide analyzer (Sievers Inst., Boulder, CO) [23]. Nitrate and nitrite (NO_x_) was measured in lavage fluid with this analyzer as previously described [24].

### 2.6. Western Blot Analysis of Tissue

Western blots were performed as described by Bratt et al. 2009 with changes indicated below. Antibodies were purchased from Santa Cruz Biotechnology, Inc. (Santa Cruz, CA) unless otherwise stated. 

The isolated airways were prepared by microdissecting the left lung lobe to obtain a preparation of the larger airways distal to the carina from the left bronchus through the third generation of conducting airway, separate from adhering parenchyma. The tissue was homogenized and processed as described by Bratt et al. [18]. Samples containing 20 *μ*g total protein were electroporated under reducing conditions and transferred to a polyvinylidene difluoride (PVDF) membrane. Membranes were incubated in 0.6 *μ*g/mL rabbit antimouse NOS1, 0.8 *μ*g/mL NOS2, 0.6 *μ*g/mL NOS3, or 0.4 *μ*g/mL *α*-Actinin IgG (used as a gell loading control) in 5% dry milk in PBS overnight at 4°C and incubated in 40 ng/mL horseradish peroxidase- (HRP-) conjugated goat antirabbit IgG (Pierce Biotechnology, Rockford, IL) in 5% milk in PBS. Bands were visualized using Immobilon western chemiluminescent HRP substrate kit (Millipore, Billerica, Massachusetts) and band intensity-assessed using the Kodak 1D version 3.5.4 scientific imaging system (Eastman Kodak Co, CT).

### 2.7. Histological Preparation

Half of the animals had their lungs fixed for histological evaluation at 30 cm pressure using 1% paraformaldehyde in PBS (pH 7.5). After 24 hours of fixation, the left lung was placed in 70% ethanol and embedded in paraffin. Lung sections of 5-um thickness were made with special attention to cutting through the larger lobar bronchi in parallel then dried at 37°C overnight.

Lung sections were deparaffinized and processed for hematoxylin and eosin (H&E) staining for evaluation of total inflammation, or NOS2-specific immunohistochemistry for semiquantitative assessment of tissue-specific NOS2 expression or Alcian Blue-Periodic Acid-Schiff (PAS) staining for quantitation of mucus-containing goblet cells.

### 2.8. NOS2 Immunohistochemistry

Left lung sections were prepared as described. Slides were incubated in 1mM ETDA, pH 7.5 at 100°C for 20 minutes to decloak antigen. Sections were processed using the R&D Systems Cell and tissue staining kit HRP-DAB System (Minneapolis, MN) for rabbit antibodies. Sections were incubated overnight at 4°C in 0.2 *μ*g/mL rabbit anti-mouse NOS2 IgG diluted in 5% goat serum in 1% BSA, 0.2 *μ*g/mL rabbit IgG (isotype control), or serum + BSA only (negative control). All other reagents were diluted as per manufacturer's instructions. Incubation times were as follows: 1 hour in biotinylated goat antirabbit secondary antibody, 30 minutes in HSS-conjugated HRP, and 15 minutes in diaminobenzidine. 

A blinded observer scored the results of the NOS2 immunohistochemical staining. A grading system of 0–10 was established prior to the grading based upon a series of prestained slide standards. The lung tissue compartments were divided into airway epithelium, smooth muscle, and macrophages and were scored under 200X power. The linear intensity grading scale used was 0—no NOS2 stain as compared to a primary antibody negative control to 10—dramatically increased NOS2 staining comparable to a lung section from an LPS-treated mouse.

### 2.9. Alcian Blue-Periodic Acid-Schiff (PAS) Staining

Left lung sections, prepared as described above, were immersed in a 1% alcian blue, 3% glacial acetic acid solution (pH2.5) for 30 minutes, rinsed in tap water, and then immersed in a 1% periodic acid solution for 7 minutes. Slides were then rinsed again in distilled water and immersed in Schiff Reagent Solution consisting in 0.45% basic fuchsin, 10% HCl, and 0.45% sodium bisulfite for 15 minutes. Slides were cleared in running tap water, counterstained using Harris' hematoxylin, and then dehydrated and mounted in cytoseal. 

Each animal was represented by a single section of lung selected for maximal visualization of the main airway. Each section had 5 randomly selected regions evaluated (two segments of the 1′ conducting airway, two segments from separate 2′ conducting airways, and one segment from a 3′ conducting airway). A minimum of 100 sequential airway epithelial cells were counted from each region and the total number of PAS positive cells per total epithelial cells was determined for each region. These regional values were then averaged to give a final PAS score per animal.

### 2.10. Statistical Analysis of Data

Results are presented as mean values ± SEM. Means were compared by *t*-test or by ANOVA, with Tukey's correction for multiple comparisons applied where appropriate. A *P*-value of .05 or less was taken to indicate significance. Analysis of the compliance and resistance changes was done by both two-way ANOVA with Bonferroni correction and by linear regression analysis, using the Prism software package (Graphpad Prism 5.0, San Diego, CA). We believe that the combination of these methods allowed for better understanding of the interaction between the effect of OVA exposure and methacholine aerosol challenge. R, a common open source statistical computing package (URL: http://www.R-project.org), was used to perform this analysis.

## 3. Results

### 3.1. Lung Inflammation in NOS Knockout Mice Exposed to Ovalbumin

**Figure 1:**
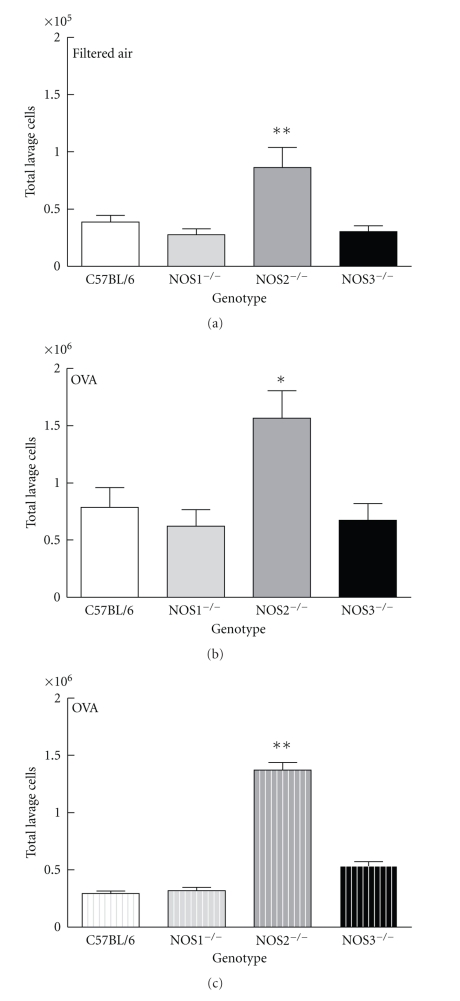
Total cells recovered by lung lavage from strains of mice exposed to filtered air (a), or 2 weeks of OVA aerosol, (b) and total eosinophils from mice exposed to 2 weeks of OVA aerosol (c). Of the filtered air-exposed mice (a), the NOS2^−/−^ mice had a greater number of cells present in their lavage (8.65 ± 6.90 × 10^4^ (*n* = 16), *P* < .01) compared to the other genotypes of mice examined (NOS1^−/−^  2.78 ± 1.71 × 10^4^ (*n* = 12), NOS3^−/−^3.03 ± 1.68 × 10^4^ (*n* = 11), C57Bl/6 3.85 ± 2.98 × 10^4^ (*n* = 27)). Of the OVA-exposed mice, the NOS2^−/−^ mice had 15.65 ± 9.93 × 10^5^ (*P* < .05, *n* = 17) cells (b), significantly more than either NOS1 (6.20 ± 5.05 × 10^5^ (*n* = 12)), NOS3 (6.7 ± 1.4 × 10^5^ (*n* = 11)), or C57BL/6 (7.86 ± 8.02 × 10^5^ (*n* = 21)) mice. Eosinophils comprised a significant proportion of the cells present in the lavage of mice exposed to OVA. NOS2^−/−^ mice exposed to OVA had 11.9 ± 1.76 × 10^5^ eosinophils (c) in lavage, which was significantly more than in NOS1 (2.4 ± 0.6 × 10^5^), NOS3 (5.3 ± 0.45 × 10^5^), or C57BL/6 (2.9 ± 3.0 × 10^5^) mice exposed to OVA. Data are presented as mean values ± SEM. * denotes *P* < .05; **P < .01 by ANOVA.

NOS2^−/−^ mice exposed only to filtered air contained 8.65 ± 6.90 × 10^4^ total cells per lavage sample (Figure 1(a)), of which 92 ± 2% were pulmonary alveolar macrophages, significantly more total cells than were found in the NOS1^−/−^, NOS3^−/−^, and C57BL/6 mouse strains exposed only to filtered air (*P* < .01). 

After two weeks of OVA exposure, the total inflammatory cell number recovered by lavage increased significantly in all strains of mice evaluated. NOS2^−/−^ mice exposed to OVA for two weeks had significantly more lung lavage cells than the NOS1^−/−^, NOS3^−/−^, or C57BL/6 mice exposed to OVA (Figure 1(b), *P* < .05). 

The normal lung lavage from a healthy mouse contains more than 90% alveolar macrophages, and our observations in this study were consistent with this finding. There were <1% eosinophils (88 ± 230 eosinophils) in all of the strains of mice tested after exposure to filtered air (Figure 1(c)). NOS2^−/−^ mice exposed to OVA had 11.90 ± 1.76 × 10^5^ eosinophils in their lavage fluid, which was significantly more than were observed in NOS1^−/−^, NOS3^−/−^, or C57BL/6 mice exposed to OVA (*P* < .01). 

There was a small (<17,000 cells) but significant increase in the number of lymphocytes in all groups of OVA-exposed mice compared to filtered air-exposed mice (data not shown), but no significant differences between the four different strains exposed to OVA. In addition, the percentage of neutrophils was <1% in all mouse strains exposed to OVA. The rest of the cells in the lung lavage fluid were macrophages. 

These results extend our previous finding that mice lacking the inducible NOS2 gene are more susceptible to allergic airway inflammation [1] than wild-type or constitutive NOS knockout mice strains.

### 3.2. Exhaled NO and NO_x_ Concentrations in NOS Knockout Mice

**Figure 2:**
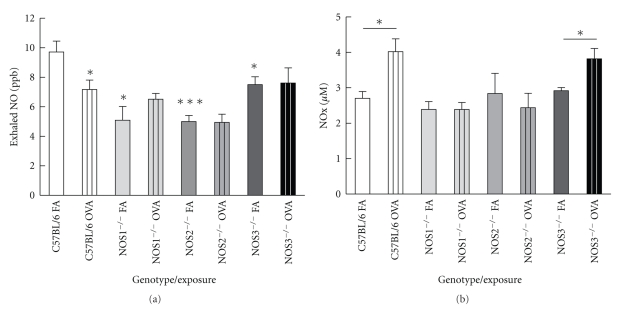
Exhaled NO (a) and lung lavage NO_x_ (b) concentrations in strains of mice exposed to filtered air or 2 weeks of OVA. NOS2^−/−^ mice exposed to filtered air have a lower exhaled NO concentration compared to C57Bl/6 mice exposed to filtered air (2.24 ± 1.7 (*n* = 15) versus 5.12 ± 4.2 (*n* = 26) ppb, *P* = .02). Air-exposed NOS2^−/−^ mice have lower exhaled NO levels compared to air-exposed C57Bl/6 mice (5.10 ± 0.41 (*n* = 8) versus 9.1 ± 0.7 (*n* = 8) ppb, *P* < .001). Air-exposed NOS1^−/−^ and NOS3^−/−^ also had significantly lower exhaled NO levels compared to the C57Bl/6 mice (*P* < .05). After exposure to OVA, there were no significant increases in the exhaled NO levels in any strain compared to their respective air-exposed group. C57Bl/6 mice exposed to OVA had a significant decrease in exhaled NO compared to their filtered air controls in this experiment. In contrast, lung lavage NO_x_ concentration (b) from NOS1^−/−^ and NOS2^−/−^ mice exposed to OVA was significantly less than C57Bl/6 mice exposed to OVA. Data are presented as means ± SEM. * denotes *P* < .05; ***P < .001 by ANOVA.

To determine which NOS isoform is responsible for the production of NO in the expired breath, we compared the concentration of exhaled NO between C57Bl/6 mice and the three NOS^−/−^ mouse strains exposed to filtered air. All NOS^−/−^ strains exposed to filtered air had similar exhaled NO concentrations (Figure 2(a)); NOS1^−/−^ (5.1 ± 1.6 ppb), NOS2^−/−^ (5.0 ± 0.5 ppb), and NOS3^−/−^ mice (7.5 ± 1.8 ppb), respectively. C57BL/6 mice exhaled a significantly greater NO concentration than NOS2^−/−^ animals (9.7 ± 0.5 versus 5.0 ± 0.5 ppb, *P* < .001). 

After exposure for two weeks to OVA aerosol, there was no significant change in exhaled NO concentrations in any of the knockout mice strains compared to their respective filtered air-exposed groups (Figure 2(a)). However, the NOS2^−/−^ animals exposed to OVA had significantly lower NO concentrations in exhaled breath than the NOS3^−/−^ mice exposed to OVA (4.9 ± 0.5 versus 7.7 ± 1.1 ppb, resp., *P* < .05). In contrast, there was a decrease in exhaled NO in the C57BL/6 mice exposed to OVA compared to their controls exposed to filtered air (9.7 ± 0.5 versus 7.2 ± 0.6 ppb, *P* < .05).

Lung lavage NO_x_ concentrations of filtered air-exposed mice (Figure 2(b)) from mice of all four strains were not significantly different from each other. After exposure to OVA, both NOS3^−/−^ and C57Bl/6 mice had significant increases in NO_x_ levels compared to their respective filtered air groups. C57BL/6 mice exposed to OVA also had significantly higher NO_x_ levels compared to NOS1^−/−^ and NOS2^−/−^ mice exposed to OVA. We examined whether NOS isoform protein expression, NO levels in exhaled breath, or NO_x_ concentrations in bronchoalveolar lavage were correlated by linear regression analysis. We found that exhaled NO levels were lower in C57Bl/6 mice after exposure to ovalbumin compared to their matched controls exposed only to filtered air. Interestingly, this result differed distinctly from our lung lavage NO_x_ data. Total nitrate/nitrite, the more stable products of NO metabolism, was increased in the C57Bl/6 mice exposed to ovalbumin. It is unclear to us why this might be a strain-related result. There is no obvious reason to believe that NO consumption is greater in lungs of inflamed wild type mice compared to others. Overall, we have some evidence that total lung NO content was increased in the mice after ovalbumin exposure. 

**Figure 3:**
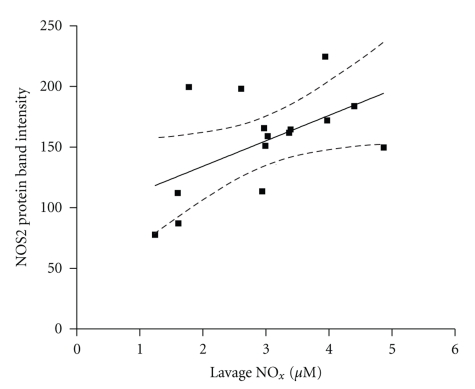
Correlation between the concentrations of NO_x_ measured in lung lavage and intensity of staining for NOS2 protein in airways of NOS3^−/−^ mice exposed to both air and OVA. Data are presented as both raw data and best fit line with 95% confidence intervals; (*m* = 18.02 ± 6.17, *P* = .01).

There was no correlation between NOS2 protein expression in the isolated airways and exhaled NO in any of the strains examined (data not shown). However, there was a significant correlation between NOS2 protein expression in the isolated airways and bronchoalveolar lavage nitrate/nitrite concentration in the filtered air and OVA-treated groups of the NOS3^−/−^ strain (band intensity versus NO_x_ concentration *m* = 18.02 ± 6.17, *r* ^2^ = 0.36, and *P* < .05, Figure 3). There were no significant correlations between NO_x_ concentration and NOS protein expression in the other mouse strains (data not shown).

### 3.3. Lung Physiology in NOS Knockout Mice

**Figure 4:**
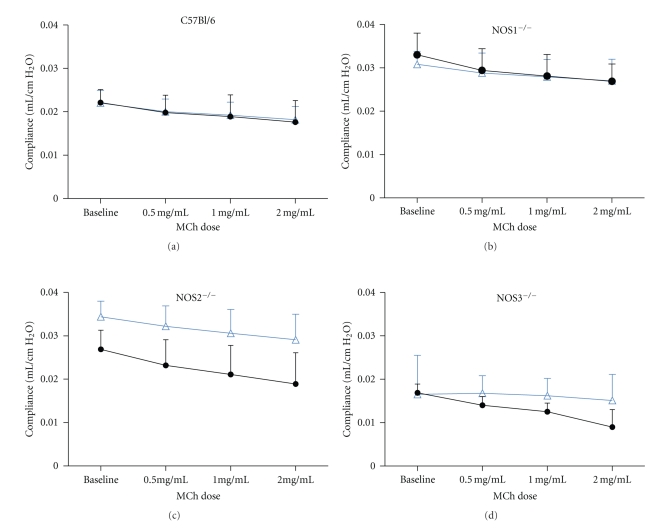
Total lung compliance in (a) C57BL/6, (b) NOS1^−/−^, (c) NOS2^−/−^, and (d) NOS3^−/−^ mice exposed to either filtered air or 2 weeks of OVA. *Symbols*: (blue) open triangles for filtered air exposure, (black) circles for OVA exposure. Lung compliance was measured at baseline and following serial doses (0, 0.5, 1.0, and 2.0 mg/mL) of nebulized methacholine (MCh). The slope of the MCh response for NOS2^−/−^ mice exposed to OVA (−0.0028 ± 0.00042, *F* = 43.56) was significantly different from that of the NOS2^−/−^ mice exposed to air (−0.0003 ± 0.00017, *F* = 3.0; *P* = .005). For NOS3^−/−^ mice, the slopes of the best fit lines for the MCh response curve for OVA-exposed mice (−0.0005 ± 0.0002, *F* = 4.627) and filtered air-exposed mice (−0.003 ± 0.0003, *F* = 95.07) were also different (*P* = .004). NOS3^−/−^ mice (both air and OVA exposed) had significantly lower lung compliance at baseline compared to all of the other strains and were most different from the NOS1^−/−^ (Cdyn: 0.017 ± 0.002 (*n* = 12) versus 0.031 ± 0.0008 (*n* = 8) mL/cmH_2_O, resp., *P* < .0001) and NOS2^−/−^ (Cdyn: 0.017 ± 0.002 versus 0.029 ± 0.0008 (*n* = 24) mL/cmH_2_O, resp., *P* = .0002).

To measure the development of airway hyperreactivity (AHR) in our model, we compared the total lung resistance and dynamic compliance at baseline and after inhalation of nebulized methacholine using serial doses of methacholine (0.5, 1.0, and 2.0 mg/mL). Lung compliance decreased significantly in NOS2^−/−^ mice exposed to OVA compared to filtered air (Figure 4(c)). The difference in lung compliance between the air and OVA-exposed NOS2^−/−^ mice was significant at each dose of methacholine (*P* < .001 for 0–2.0 mg/mL methacholine). The slope of the MCh response by linear regression analysis for NOS2^−/−^ mice exposed to OVA (−2.6 ± 0.27 × 10^−3^, *F* = 96.35) was significantly different from that of the NOS2^−/−^ mice exposed to air (−1.8 ± 0.12 × 10^−3^, *F* = 226.9; *P* < .05). Measured lung compliance after exposure to methacholine also significantly differed for NOS3^−/−^ mice exposed to OVA versus air (Figure 4(d)). The slopes of the best fit lines by linear regression analysis for the methacholine dose-response curve for OVA-exposed NOS3^−/−^ mice, −0.5 ± 0.2 × 10^−3^, *F* = 4.627, and for filtered air-exposed NOS3^−/−^ mice was −3.0 ± 0.3 × 10, *F* = 95.07  (*P* = .004). Neither the C57BL/6 nor the NOS1^−/−^ mice demonstrated significant decreases in lung compliance with methacholine challenge after exposure to OVA (Figures 4(a) and 4(b)). 

**Figure 5:**
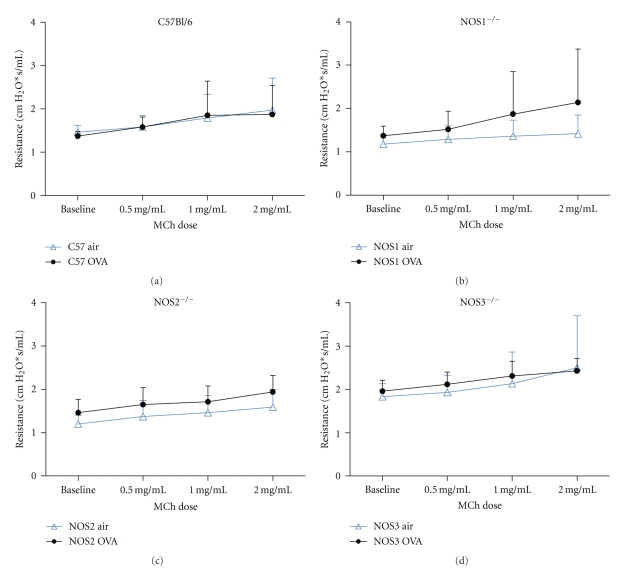
Total lung resistance in (a) C57BL/6, (b) NOS1^−/−^, (C) NOS2^−/−^, and (D) NOS3^−/−^ mice exposed to either filtered air or 2 weeks of OVA. *Symbols*: (blue) open triangles for filtered air exposure, (black) circles for OVA exposure. Lung resistance was measured at baseline and following serial doses (0.5–2.0 mg/mL) of nebulized methacholine. NOS1^−/−^ mice exposed to OVA (slope = 0.266 ± 0.027, *F* = 94.09) had a significantly greater increase in lung resistance compared to the same strain of mice exposed to filtered air (slope = 0.079 ± 0.008, *F* = 93.15; *P* = .002). Air- and OVA-exposed NOS2^−/−^ mice had similar increases in lung resistance (slopes = 0.12 ± 0.01 and .015 ± 0.02, resp.), but their intercepts were significantly different (*y* intercept 1.09 ± 0.02 versus 1.3 ± .0.06, resp., *P* = .0001).

Lung resistance measurements from the four different strains of mice examined did not show significant differences when analyzed by two-way ANOVA (Figures 5(a)–5(d)) but differed significantly compared to their corresponding filtered air exposures when analyzed by linear regression analysis. Upon challenge with increasing doses of MCh, NOS1^−/−^ mice exposed to OVA (slope = 0.266 ± 0.027, *F* = 94.09) had a significantly greater increase in lung resistance compared to the same strain of mice exposed to filtered air (slope = 0.079 ± 0.008, *F* = 93.15; *P* = .002). Air- and OVA-exposed NOS2^−/−^ mice had similar increases in lung resistance upon MCh challenge testing (slopes = 0.12 ± 0.01 and 0.15 ± 0.02, resp.). These results suggest that these strains of mice behaved differently than the NOS1^−/−^ or NOS3^−/−^ mice after their respective exposures, which could reflect differences in their inflammatory responses, differences in airway remodeling, or both factors.

### 3.4. NOS Protein Content in Airways Isolated from NOS Knockout Mice

**Figure 6:**
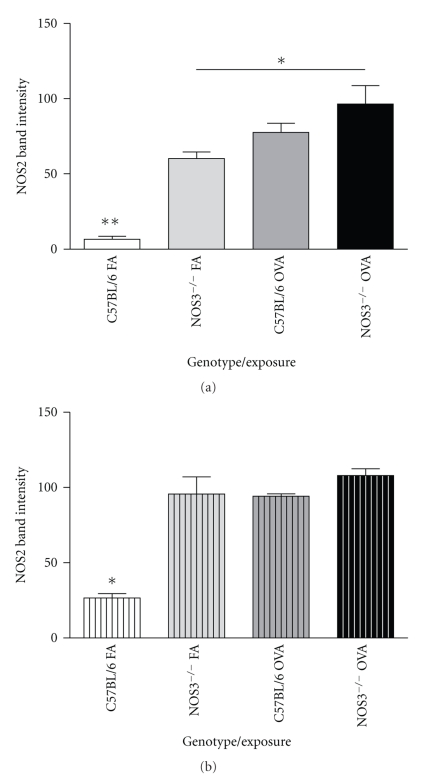
Relative band intensity of NOS2 protein by Western blot in (a) NOS1^−/−^ and (b) NOS3^−/−^ mice. NOS1^−/−^ animals showed an upregulation of inducible NOS2 in air control animals and maintained the pattern of NOS2 upregulation in response to OVA treatment. NOS3^−/−^animals also showed a significant upregulation of NOS2 in filtered air-exposed animals, but no change in NOS2 in response to OVA treatment. Data are expressed as mean values ± SEM (*n* = 4). * denotes *P* < .05; **P < .01 by ANOVA.

**Figure 7:**
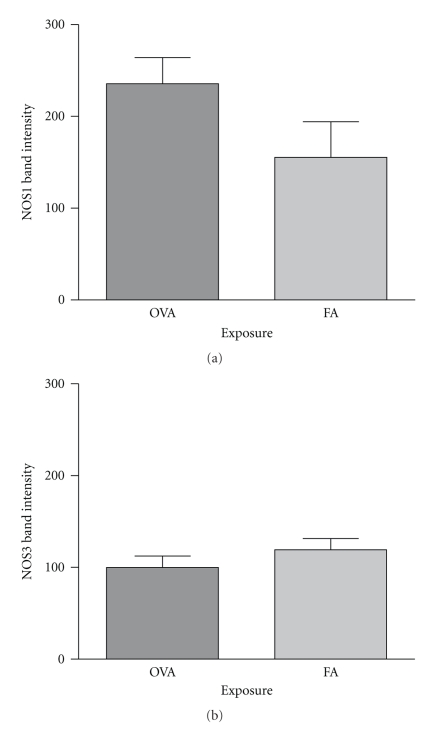
Relative band intensity of staining of (a) NOS1 protein and (b) NOS3 protein by Western blot in NOS2^−/−^ mice. No significant change is in the protein levels of either NOS1 or NOS3 in airways of mice exposed to filtered air or OVA. Data are expressed as mean values ± SEM (*n* = 4 each).

The relative amount of the different NOS isoforms in airways isolated from each of the mouse strains was assessed using western blot band intensity analysis. Protein blots of airways from both the NOS1^−/−^ (Figure 6(a)) and NOS3^−/−^ (Figure 6(b)) mice exposed to filtered air showed a higher NOS2 protein expression than the C57Bl/6 controls (NOS1^−/−^  60.2 ± 10.7 versus wt C57 Bl/6 6.5 ± 3.6, *P* < .01) and (NOS3^−/−^95.6 ± 28.1 versus wt C57 Bl/6 26.6 ± 5.1, *P* < .05). Upon OVA exposure, the NOS2 expression in NOS1^−/−^ animals was further upregulated (96.5 ± 30.1 (OVA) versus 60.2 ± 10.7 (FA), *P* = .019), while NOS3^−/−^ animals (Figure 6(b)) showed no change in NOS2 with OVA treatment compared to their filtered air controls. In contrast, the NOS2^−/−^ mice (Figures 7(a) and 7(b)) showed no significant change in the protein levels of either NOS1 or NOS3 in the airway samples from mice that were exposed to filtered air or OVA.

### 3.5. Tissue-Specific NOS2 Protein Content in NOS Knockout Mice

**Figure 8:**
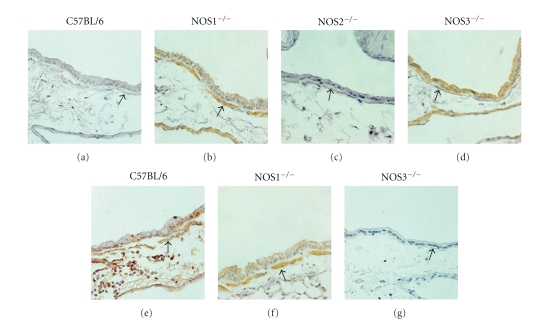
Immunohistochemical stain of NOS2 protein in 5 *μ*m thick left lung sections from C57Bl/6 (a) filtered air-exposed and (e) OVA-exposed mice, NOS1^−/−^ (b) filtered air-exposed and (f) OVA-exposed mice, NOS2^−/−^ (c) filtered air-exposed mice, and NOS3^−/−^ (d) filtered air-exposed and (g) OVA-exposed mice. Airway smooth muscle layer is indicated by arrow. Images were taken at 400× magnification.

**Figure 9:**
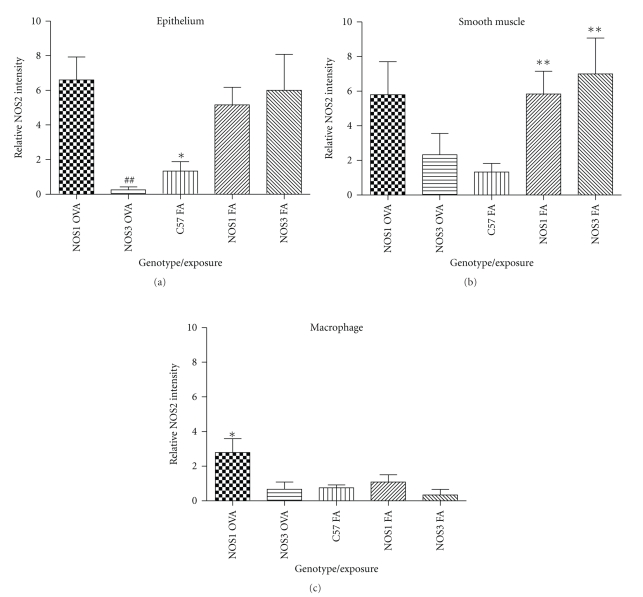
Semiquantitative analysis of NOS2 staining intensity from NOS1^−/−^ and NOS3^−/−^ mouse strains exposed to filtered air or OVA. Immunohistochemical staining intensity and consistency of stain in the (a) airway epithelium, (b) smooth muscle of the airways and vasculature, and (c) macrophage populations were scored on a scale of 0–10. Filtered air-exposed NOS1^−/−^ and NOS3^−/−^ mice displayed uniform increases in NOS2 protein staining limited to the smooth muscle of airways and vasculature and the airway epithelium with no change in macrophages. OVA-exposed NOS3^−/−^ mice had a significant reduction in NOS2 staining in the airway epithelium and smooth muscle compared to their filtered air-exposed counterparts. In contrast, OVA-exposed NOS1^−/−^ mice maintained NOS2 protein staining in the airway epithelium and a significant increase in NOS2 in the macrophage population. Data are presented as mean values ± SEM (*n* = 5-6); * denotes *P* < .05; **P < .01 by ANOVA.

We analyzed smooth muscle tissue, airway epithelium, and macrophage populations to evaluate differences in NOS2 protein content between these three lung tissue compartments. Consistent with the western blot data derived from the isolated airways, NOS1^−/−^ and NOS3^−/−^ mice exposed to filtered air had a greater NOS2 protein content than C57Bl/6 mice exposed to filtered air (Figures 8 and 9). Quantitative assessment by immunohistochemistry of these air-exposed mice showed that increases in NOS2 protein content were limited to the airway epithelium (NOS1^−/−^ versus C57BL/6: *P* < .05 and NOS3^−/−^ versus C57Bl/6: *P* < .05) and the smooth muscle of the airways and vasculature (NOS1^−/−^ versus C57BL/6: *P* < .01 and NOS3^−/−^ versus C57Bl/6: *P* < .01), but no change in NOS2^−/−^ content was detected in the tissue macrophage population (Figure 9). 

OVA exposure significantly reduced total NOS2 protein in the airway epithelium and smooth muscle tissue compared to the filtered air-exposed animals in the NOS3^−/−^ mice (Figures 9(a) and 9(b), *P* < .01). In contrast to the NOS3^−/−^ mice exposed to OVA, the NOS1^−/−^ mice exposed to OVA maintained the NOS2 protein content in the airway epithelium and also showed significant increases in NOS2 protein content in the macrophage population (Figures 9(a) and 9(c), *P* < .05 compared to OVA-exposed NOS3^−/−^ and filtered air-exposed NOS1^−/−^ mice).

### 3.6. Goblet Cell Metaplasia in NOS Knockout Mice

**Figure 10:**
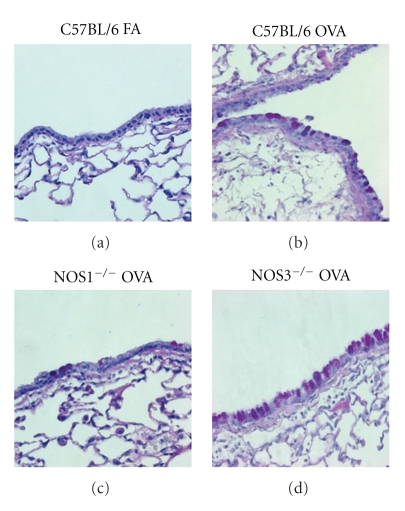
PAS staining of 5 *μ*m-thick left lobe lung sections from (a) C57Bl/6 filtered air-exposed, (b) C57Bl/6 OVA-exposed, (c) NOS1^−/−^ OVA-exposed, and (d) NOS3^−/−^ OVA-exposed mice. Images were taken at 400× magnification.

**Figure 11:**
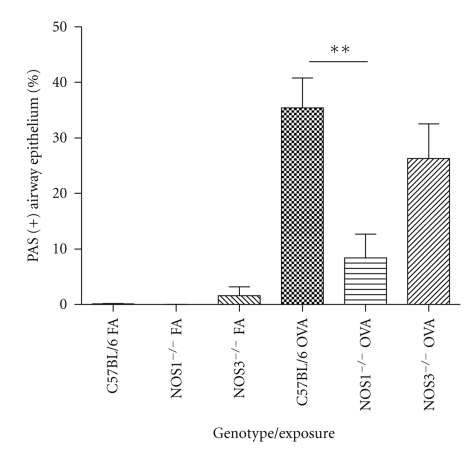
Percentage PAS positive cells present in the airway epithelium from C57Bl/6, NOS1^−/−^, and NOS3^−/−^ genotype mice exposed to filtered air or OVA. Filtered air-exposed mice displayed an average of 0.45% PAS positive cells with no difference between the three groups. OVA-exposed NOS1^−/−^ mice had significantly fewer PAS positive-stained cells compared to the OVA-exposed C57Bl/6. Data are presented as mean values±SEM; ** denotes *P* < .01 by ANOVA.

To determine if the decrease in NOS2 protein content in the airway epithelium was due to the replacement of ciliated epithelium with mucus-producing goblet cells in the NOS3^−/−^ animals exposed to OVA, we used the Periodic Acid-Schiff (PAS) to stain for mucus-containing cells in the airway epithelium and determined the overall ratio of mucus-containing cells to total cell population by cell counting. All of the filtered air-exposed groups contained essentially no mucus-producing cells in the airways (see, e.g., C57BL/6 animals; Figure 10(a)). In contrast, exposure to OVA significantly increased the number of mucus-containing cells in all three strains of mice tested (Figures 10(b)–10(d)). We observed that 9–37% of the total cell population of the upper airway epithelium was mucus-producing cells, and that the observed value depended on the genotype (Figure 11) with NOS1^−/−^ mice exposed to OVA having significantly fewer PAS positive-stained cells compared to the OVA-exposed C57Bl/6 (*P* < .01) or NOS3^−/−^  (*P* < .05) mice.

## 4. Discussion

### 4.1. Anti-Inflammatory Role of NOS2 and Its Effects on Lung Function

NO modulates pulmonary vascular tone, non-adrenergic non-cholinergic mediated bronchodilation, the lung inflammatory response [25], and apoptosis [26–29]. In previous work using an NOS2^−/−^ strain, we observed increased airway inflammation as compared to wild-type mice exposed only to air, and especially upon exposure to OVA [1]. We concluded that the ability to upregulate active NOS2 enzyme in response to OVA exposure is necessary for the anti-inflammatory effect imparted by NO. However, these earlier findings could not define the mechanistic basis for these observations, for example, whether NOS2 was itself the required source for NO in allergen-induced airway inflammation or whether the effects were caused by increased L-arginine turnover by the other NOS isoforms or the reconversion of oxidization products, nitrate and nitrite, into NO. To attempt to answer these questions, we examined the effects of deleting individual NOS isoforms on the overall dynamic of total lung NO balance in allergic airway disease. 

Measurements of exhaled NO in the various mouse strains tested focused our attention on NOS2 as the most likely source of NO in exhaled air from mice exposed to ovalbumin (Figure 3). Examination of the correlation between NOS2 protein expression in isolated airways and total concentration of NO_x_ in the lung lavage fluid showed that the NOS3^−/−^ strain was the only strain studied, where NOS2 protein expression was tightly coupled to NO_x_ production, and such coupling was observed in the NOS3^−/−^ mice exposed to either air or ovalbumin. Steudel et al. [30] examined exhaled NO from NOS1^−/−^, NOS2^−/−^, and NOS3^−/−^ mice. The NOS2^−/−^ mice showed decreased exhaled breath NO as compared to control animals. In contrast, the NOS1^−/−^ and NOS3^−/−^ mice actually exhaled more NO than their wild-type counterparts. The upregulation of NOS2 we observed in the two constitutive NOS knockout strains may explain this finding. 

In previous work using the NOS2^−/−^ strain [18], we observed increased numbers of cells in the lavage fluid (predominantly macrophages), which raised the question of whether deletion of either of the other individual NOS isoforms (NOS1 and NOS3) would have similar effects on the lavagable alveolar macrophage population. Because we hypothesized that there would be an upregulation of NOS2 in the constitutive knockouts, we expected to find no significant increase in total lung cells in lavage fluid from the NOS1 and NOS3^−/−^ animals exposed to filtered air. Our results were consistent with this hypothesis, as we found similar BAL total cell counts in the constitutive knockout strains and the C57Bl/6 mice. We conclude that the deletion of individual NOS isoforms affects NO output by specific isoforms, but that the inability to normalize the total lung NO concentration by upregulating NOS2 results in an increase in the recruitment of bone marrow-derived cells as illustrated by an increase in the population of lavagable macrophages only in the NOS2^−/−^ mice. 

In this study, NOS1^−/−^ animals exposed to filtered air demonstrated a significant change in lung compliance and resistance upon challenge with methacholine. With increased NOS2 protein expression induced by OVA exposure, this reactivity appeared to be diminished. We also saw a significant decrease in the respiratory system compliance of NOS2^−/−^ mice exposed to OVA compared to their counterparts exposed to filtered air. We interpret these findings to suggest that changes in lung compliance in this model directly reflect lung inflammation. Only the NOS1^−/−^ animals exposed to OVA demonstrated a significant decrease in lung compliance associated with a concomitant increase in lung resistance that reflects a bronchoconstrictive response. These data suggest that NO from a specific enzyme source—in this case from NOS1 in the subepithelial smooth muscle and/or the epithelium—is responsible for the bronchodilatory properties of NO, but that the induction of NOS2 may be sufficient to mitigate some aspects of AHR. 

Samb et al. [31] and Maarsingh et al. [32] attribute decreased NOS1 activity in pulmonary and tracheal smooth muscle after OVA challenge in guinea pigs to airway hyperresponsiveness, and our data are consistent with these results. De Sanctis et al. also examined changes in inflammatory response in the NOS2^−/−^ strain compared to the C57Bl/6 strain but observed no significant increase in total inflammatory cell number [33]. Although our current and previous observations appear to conflict with the findings of De Sanctis, substantial variations in the exposure protocol may have caused the differing outcomes.

### 4.2. Regulation of NOS Isoforms and Consequences in Normal Mice

NOS2 is capable of producing one thousand times more NO than either of the constitutive isoforms [34] and has multiple levels of regulation that are dependent upon NO concentration [10, 35–41]. We hypothesized that under normal noninflammatory conditions, deletion of the NOS1 or NOS3 gene would upregulate NOS2 protein expression. Knocking out individual constitutive NOS isoforms would lower overall cellular NO concentration, resulting in increased NOS2 expression in order to maintain a minimal total lung NO concentration. To test this hypothesis, we measured lung NO and NO_x_ concentrations, total airway NOS protein content, and localized the expression of NOS2 protein in the four strains of mice (C57Bl/6 wild-type strain and NOS1^−/−^, NOS2^−/−^, and NOS3^−/−^) exposed to filtered air only. 

The constitutive NOS knockout strains, NOS1^−/−^ and NOS3^−/−^, exposed to filtered air showed significant upregulation of NOS2 protein expression compared to the C57Bl/6 wild-type control. As predicted, the NOS1^−/−^ mice showed no change in NOS3 expression and NOS3^−/−^ mice showed no change in NOS1 expression. These results support our hypothesis that “NO homeostasis” is established by maintaining a concentration of available total NO independent of NOS isoform origin.

The NOS2^−/−^ strain showed no significant increase in protein expression of either of the two constitutively expressed NOS isoforms (NOS1 or NOS3). These results indicate different upstream regulatory mechanisms inducing gene expression and potential translational control of the two constitutive isoforms and indicate that the control of constitutive NOS expression is not NO dependent. Thus, we conclude that at baseline, NOS2 enzyme production can be induced to maintain total lung NO homeostasis via a mechanism that is capable of detecting decreased concentrations of cellular NO and activating NOS2 expression. This conclusion is in accordance with observations by Cook et al. [10], who noted increased NOS2 expression and exhaled NO concentrations in NOS3^−/−^ mice. 

Immunohistochemical analysis of whole lung sections identified the cellular origins of NOS2 expression in the filtered air-exposed genotypes. Consistent with the western blot results, the C57Bl/6 mice exposed to filtered air showed low expression of NOS2 protein, with only light staining present in the smooth muscle and airway epithelium. In contrast, both the NOS1^−/−^ and NOS3^−/−^ animals displayed a significant increase in NOS2 protein expression compared to the C57Bl/6 mice by western blot analysis, and these increases were limited to the airway epithelium and smooth muscle tissue of the airway and vasculature (See Figures 8-9). As the airway epithelium constitutively expresses both NOS1 and NOS3 and is capable of upregulating NOS2 under inflammatory conditions, this result implies a tight regulatory control of NO homeostasis in these particular cell types. The smooth muscle tissue of the lung expresses low levels of NOS2 protein in the C57Bl/6 mice and may benefit from vectorial production of NO derived from adjacent epithelial and endothelial cells. As localized NO concentrations decrease, NOS2 expression may be necessary to maintain airway and vascular tone under basal conditions.

### 4.3. Regulation of NOS Isoforms and Consequences in Mice Exposed to Ovalbumin

Based upon recent studies by Vo et al. [35] and Gobeil Jr. et al [17], which suggest that induction of NOS2 gene expression by NF-*κ*B is dependent upon NOS3 activity (Figure 8), we hypothesized that exposure to ovalbumin would inhibit the increase in NOS2 protein expression in the NOS3^−/−^ mice.

We examined the upregulation of NOS2 in NOS1 and NOS3^−/−^ mice exposed to OVA to determine if the NO-dependent mechanism to induce NOS2 expression is isoform specific, that is, derived solely from the NOS3 isoform. If the OVA-induced increase in NOS2 expression is driven by the NOS3, we would expect no significant increase in NOS2 protein expression in the NOS3^−/−^ animals while we would expect an increase in NOS2 protein expression in the NOS1^−/−^ animals. In our study, the patterns of NOS2 expression in the OVA-exposed NOS1^−/−^ and NOS3^−/−^ animals were consistent with this mechanism being NOS3 specific. In fact, we observed a surprising lack of NOS2 expression in the lungs of NOS3^−/−^ animals overall by immunohistochemistry. 

Analysis of NOS2 expression in the NOS1^−/−^ and NOS3^−/−^ lung compartments of mice exposed to OVA indicated a significant reduction of NOS2 in the airway epithelium and smooth muscle compartments of the NOS3^−/−^ mice only, with NOS1^−/−^ mice maintaining NOS2 protein expression in both compartments, including upregulation of macrophage NOS2 expression. While examining NOS2 protein content in the airway epithelium, we observed a difference in the cellular population of the airway epithelium between the different mouse genotypes with exposure to OVA that may have contributed to changes in NOS2 protein content. One limitation of our study is that we used NOS gene knockout strains only and did not use overexpressing mice to confirm our findings. However, we are encouraged by the complimentary data of Ten Broeke and colleagues. They found that transgenic mice overexpressing NOS3 increased NO production in the lungs, decreased lung lavage inflammatory cell counts, and improved airway hyperresponsiveness compared to littermate controls after exposure to ovalbumin. One possible explanation for the seeming overlap between Ten Broeke's results and ours is the interrelationship among the NOSs in the lung [19]. NOS3 expression appears to partly regulate NOS2 expression and lung NO content. This suggests that important feedback mechanisms to NOS3 regulate much of the measurable NO that is produced in the lung in response to allergen. 

The NOS 1^−/−^ mice had a significantly reduced number of goblet cells present compared to the other three strains. NOS1^−/−^ mice exposed to OVA also had reduced airway reactivity and airway inflammation compared to the other strains, which may indicate roles for NOS1 in inflammatory cell recruitment signaling and, potentially, airway epithelial cell apoptosis. In addition to its effects on airway hyperreactivity, NO has pro- and antiapoptotic effects that are concentration and cellular compartment dependent. The NOS1 isoform is also localized to the mitochondria (mtNOS) and despite the ubiquitous nature of NO in the lung during inflammation, deletion of the NOS1/mtNOS isoform may contribute to the reversal of airway goblet cell metaplasia seen in the other isoform knockout and C57Bl/6 strains exposed to ovalbumin. 

Although we were able to identify increased goblet cell metaplasia in the airways of NOS3^−/−^ mice exposed to ovalbumin as compared to NOS1^−/−^ mice, this change in epithelial cell content did not account for the lack of NOS2 protein in the remaining intact epithelial cells. The cells that were PAS-negative still comprised 50% of the airway cell population. In addition, the lack of NOS2 protein in the intact smooth muscle of the airways and vasculature of the NOS3^−/−^ mice was also noteworthy. 

Though our hypothesis indicates that knockout of NOS3 results in an inability to signal for increased NOS2 expression upon allergen exposure, an alternative explanation exists for these results that we could not address within the scope of our studies in intact animals. NF-*κ*B-dependent transcription can also be affected by nitrosation of the Rel and p50 components of the active NF-*κ*B complex. In high NO environments such as cell culture medium with high concentrations of NO donor compounds added, cysteine residues of both p65 and p50 have been shown to be nitrosated, resulting in reduced DNA-binding capacity [35, 39–41] (Figure 9). Increased NO production by NOS2 under inflammatory conditions may be sufficient to nitrosate these residues resulting in the activation of this negative feedback loop.

### 4.4. Potential Alternative Sources of NO in the Lungs of the Mice Exposed to Ovalbumin

Despite the lack of NOS2 in the lungs of NOS3^−/−^ animals exposed to OVA, increases in BAL NO_x_ concentrations in the NOS3^−/−^ mice may indicate an accumulation of NO metabolites in the lung from another source. There is increasing evidence suggesting that nitrite serves as a bioavailable pool of NO to act as a vasodilator, as deoxyhemoglobin may have nitrite reductase activity, promoting this conversion [6]. While we did not measure the arterial oxygen saturation of our mice during this experiment, it is possible that the ventilation-perfusion mismatching as a result of dense inflammatory infiltrate and/or methacholine challenge could lead to regional hypoxemia and have an effect on NO_x_ to NO conversion. However, we did not see changes in either NO or NO_x_ concentrations in NOS2^−/−^ animals after exposure to OVA that would be consistent with this pathway. In addition, it has been theorized that alterations in the pH of the airway lining fluid as a result of inflammatory cell influx and oxidative damage may result in nitrite conversion to NO. Both the NOS3^−/−^ and NOS2^−/−^ mice had more inflammatory cells in their BAL fluid but their exhaled NO concentrations were relatively unaffected.

## 5. Conclusions

Examination of individual NOS isoform knockout and C56Bl/6 strains exposed to filtered air supports the hypothesis that there is an NOS-dependent mechanism in the cell that maintains a “baseline” NO production in both airway epithelium and airway and vascular smooth muscle and is unable to distinguish between NOS isoforms of origin. Under inflammatory conditions, the expression of the NOS2 isoform is essential for reducing lung inflammation and may also contribute to the normalization of airway reactivity. NOS3 isoform activity is essential for the upregulation of NOS2 in response to ovalbumin exposure. In contrast, NOS2 can be expressed independently of NOS1 activity in the inflamed lung, but NOS1 activity contributes to goblet cell metaplasia in the airways of ovalbumin-exposed animals.
